# Assessment of the antimicrobial efficacy of probiotics, biosynthesized silver nanoparticles, and their combination with physical irradiations against cattle endometritis pathogens

**DOI:** 10.1038/s41598-025-18623-5

**Published:** 2025-09-17

**Authors:** Noha E. AbdElhafeez, Salama M. El-Darier, Tatiana N. Gryazneva, Hussein A. Motaweh, Samy A. El-Aassar, Aliaa M. El-Borai

**Affiliations:** 1 Botany and Microbiology Department, Faculty of Science, Alexandria University, Alexandria, Egypt; 2 Microbiology Department, Medicine and Biotechnology- Mva Named, After K. I. Skryabin, Moscow State Academy of Veterinary, Moscow, Russia; 3 Physics Department, Faculty of Science, Damanhour University, El-Beheira, Egypt

**Keywords:** Probiotics, Green synthesis of silver nanoparticles, Multidrug-resistant bacteria, Fungal pathogens, Antimicrobial effect, Biophysics, Portable irradiation device, Vaginal gel, Biotechnology, Microbiology

## Abstract

Endometritis is a postpartum uterine inflammation caused by bacterial infection often linked to complications such as abortion. The current study aims to manage microbial endometritis in cattle using the antimicrobial effects of probiotics, biosynthesized silver nanoparticles (AgNPs), and biophysical factors. The antimicrobial effects of some selected commercial *Bacillus* probiotic strains -divided into seven groups and biosynthesized AgNPs (1–5 mM) against various pathogenic strains were investigated. Experiments included AgNPs characterization (SEM/EDX, UV–Vis, and FTIR), and disc diffusion assays. The impact of UV, IR, and 50 Hz sinusoidal EMF at 5 mT on pathogen viability; both alone and in combination with AgNPs; was assessed followed by ultrastructural analysis (TEM). Group 1 (G1) probiotics showed the highest inhibition (16.57 ± 5.59 mm), especially against *E. coli* and *P. aeruginosa*. AgNPs (5 mM) synthesized from G1 achieved maximum antimicrobial activity (up to 52 mm for *E. coli*), with IR + AgNPs reducing bacterial counts by over 75%. In contrast, UV and EMF treatments, especially when combined with AgNPs, unexpectedly increased fungal growth. This study highlights the potent antimicrobial effects of probiotics and biosynthesized AgNPs, with the combination of IR and AgNPs demonstrating the strongest inhibitory activity, particularly against bacteria. In contrast, UV and EMF treatments were less effective and, in some cases, appeared to promote fungal growth.

**Supplementary Information:**

The online version contains supplementary material available at 10.1038/s41598-025-18623-5.

## Introduction

Endometritis is a common postpartum uterine disease in cattle that significantly impacts reproductive performance and productivity. Its severity depends on bacterial load, pathogen virulence and the immune status of cow with risk factors including metabolic disorders, abnormal calving, parity and retained placenta^1^. Recent studies have shown that the pregnant uterus is not sterile, with over 200 bacterial species identified postpartum. Moreover, bacteriological tests confirm infection but do not always indicate inflammation^2^. In light of the resistance exhibited by specific bacteria to conventional treatments for endometritis, it is imperative to investigate alternative therapeutic options for this condition, particularly due to its significant implications and the economic repercussions linked to elevated infection rates in cattle^3^.

Probiotics refer to the diverse microbial communities within the human body which play a crucial role in regulating various metabolic processes^4^. Probiotics, particularly strains from *Lactobacillus*, *Bifidobacterium*, *Bacillus*, and *Pediococcus*, play an important role in host metabolism and immune defense by inhibiting pathogenic microbes^5^. In addition, probiotic bacteria are now being investigated for their ability to synthesize nanoparticles for therapeutic applications^6^. *Bacillus* species are widely used in probiotic formulations and are valued for their environmental stability and functional properties including antimicrobial and antioxidant activity^7^.

Nowadays, nanoparticles are gaining interest as drug and nutrient delivery systems, reproductive aids, and antimicrobial alternatives^8^. Metallic nanoparticles, especially silver nanoparticles (AgNPs), offer advantages such as tunable optical properties, thermal stability, and antimicrobial efficacy^9^. AgNPs have revealed strong activity against bacteria, fungi, viruses and also antibiotic-resistant strains^8,10^. While AgNPs can be synthesized by physical, chemical, or biological methods, green synthesis is preferred due to the limitations of conventional techniques^11^.

Radiation types such as ultraviolet (UV), infrared (IR), and electromagnetic fields (EMF) have established varying degrees of antimicrobial activity. UV radiation, particularly UV-C (ranging from 200–280 nm), is highly effective in deactivating microorganisms by inducing DNA damage, disrupting replication and transcription and generating reactive oxygen species (ROS)^12^. Microbial sensitivity to UV varies by species, cell wall composition, and physiological state, with Gram-negative bacteria being more susceptible than Gram-positive and fungal organisms^13^. EMFs, especially low-frequency fields (e.g., 50 Hz), can alter microbial growth, viability, and antibiotic susceptibility, potentially by inducing oxidative stress or modifying gene expression^14^. However, results are mixed, and effects may depend on field strength, exposure time, and microbial species. IR radiation, particularly in the near-IR range, has shown potential antimicrobial activity through mild thermal effects that disrupt protein structure and cell membranes. Additionally, IR enhances wound healing, modulates inflammation, and may synergize with other treatments^15^.

The current study aims to control microbial endometritis in cattle by evaluating the antimicrobial potential of selected commercial probiotics, biosynthesized silver nanoparticles, and various types of radiation. Moreover, it investigates the combined effect of silver nanoparticles and radiation on pathogenic strains associated with the disease.

## Materials and methods

### Studied microorganisms and culture media

Commercial probiotic strains (group 1 (G1): *Bacillus subtilis* VKM B-2287 + *Bacillus licheniformis* VKM B-2414; group 2 (G2): *Bacillus subtilis* VKM B-10172 + *Bacillus licheniformis* VKM B-10135; group 3 (G3): *Bacillus cereus* VKM B-5832) and pathogenic test microorganisms (*Escherichia coli* VKM B-1776, *Pseudomonas aeruginosa* VKM B-190155, *Staphylococcus aureus* VKM B-7526, *Streptococcus agalactiae* VKM B-2603, *Candida albicans* VKM 303,903, *Aspergillus niger* VKM F-2259, *Aspergillus fumigatus* VKM F-1863) were obtained from the All-Russian Collection of Microorganisms (VKM). Bacteria were cultured in meat peptone agar at 37°C for 24 h; *S. agalactiae* was grown on meat peptone agar supplemented with 5% defibrinated sheep blood. Fungi were incubated in Sabouraud dextrose agar at 27°C for 96 h. All strains were maintained at 4°C and sub-cultured every 2–3 weeks. For antimicrobial assays, liquid cultures were prepared in corresponding broths. Mueller Hinton Agar (MHA) and Mueller–Hinton agar with 2% glucose and methylene blue (MH-GMB) were used for antibacterial and antifungal susceptibility testing, respectively^16^.

### Microbiological media preparation

Meat peptone media (agar and broth) and Sabouraud dextrose media (agar and broth) were prepared according to standard protocols for bacterial and fungal cultivation, respectively. Mueller–Hinton agar (MHA) and Mueller–Hinton glucose methylene blue (MH-GMB) media were prepared for antimicrobial susceptibility testing. All media were adjusted to pH 7.0 and sterilized by autoclaving at 121 °C for 15 min. For *Streptococcus agalactiae*, MHA was supplemented with 5% defibrinated sheep blood after autoclaving.

### Preparation of probiotic supernatants

Probiotic strains (G1, G2, G3) were cultured in meat peptone broth (48h, 37°C, 150 rpm shaking). Supernatants were collected via centrifugation (5,000 rpm, 10 min). Seven probiotic groups were prepared by combining equal volumes of supernatants: G1, G2, G3, G4 (G1 + G3), G5 (G2 + G3), G6 (G1 + G2), and G7 (G1 + G2 + G3). These were used for antimicrobial and AgNPs biosynthesis studies^17^.

### Biosynthesis of silver nanoparticles using probiotics

AgNPs were synthesized by mixing 50 mL of 1–5 mM AgNO_3_ aqueous solutions with 50 mL of probiotic supernatants (G1-G7) in 250 mL Erlenmeyer flasks. Mixtures were incubated statically (40°C, 5 days, dark). Controls (AgNO3 only, pure supernatant) verified probiotic involvement^17,18^. The mixtures were examined for silver nanoparticles biosynthesis, with size and shape determination, and then used for the evaluation of antimicrobial activity.

### Characterization of silver nanoparticles

AgNPs size, shape, and elemental composition were determined by Energy Dispersive Spectroscopy (SEM/EDX) using a TESCAN LYRA 3 instrument (Skobeltsyn Institute of Nuclear Physics, Moscow State University). Samples were gold-coated for SEM imaging (5–20 kV). EDX (15–20 kV) confirmed silver presence. UV–Vis spectroscopy was conducted using a METTLER TOLEDO UV5 NANO Spectrophotometer (Institute of Solid State Physics, Russian Academy of Sciences, Chernogolovka). AgNPs were characterized by UV-spectra (250–600 nm). Purified nanoparticles were obtained by centrifugation (15,000 rpm, 2–3 cycles), filtration, and drying, with dried powder further analyzed by Philips XL-30 EDX. Fourier-transform infrared (FTIR) spectroscopy was used to identify the biomolecules involved in capping and stabilizing the biosynthesized AgNPs. Ten milligrams of dried AgNPs were mixed thoroughly with ≥ 99% spectroscopic-grade potassium bromide (KBr), and then compressed into a translucent pellet. FTIR spectra were recorded in transmission mode using a PerkinElmer Spectrum IR instrument (Version 10.6.0) across the range of 4000–450 cm⁻^1^ at a resolution of 4 cm⁻^1^ ^19,20^.

### Antimicrobial activity assays

The disc diffusion assay was performed on 90 mm Petri plates. Media were seeded with 100 µL pathogen suspensions (10⁸ CFU/mL, OD 600 nm). Sterilized 5 mm filter paper discs, dipped in test samples, were placed on inoculated agar^21^. The inhibition zones measured using calibrated digital calipers to ensure accuracy. All tests were done with equal agar thickness (4 ± 0.2 mm). Positive controls included Ampicillin, Chloramphenicol, Ciprofloxacin (antibacterial), and Amphotericin B, Clotrimazole, Nystatin (antifungal). Negative controls were included using cell-free probiotic filtrate or 1–5 mM AgNO_3_ for probiotic extracts or AgNPs synthesized by probiotics groups, respectively. Plates were incubated (24 h, 37°C for bacteria; 48–96 h, 28°C for fungi) in triplicates. To confirm susceptibility testing standard antibiotic discs were determined against all pathogenic agents.

Inhibition zones were measured in mm; values < 7 mm indicated no activity^22,23^.

### Determination of minimum inhibitory concentrations (MICs)

The MIC of AgNPs (from 5 mM AgNO₃ synthesized by G1 probiotics) was determined by agar dilution. Pathogen suspensions were standardized to 0.5 McFarland (≈10⁸ CFU/mL). AgNPs dilutions (2 mL) were added to 18 mL of Mueller–Hinton agar (cooled to 50°C), poured into Petri dishes, and allowed to solidify. Then, 100 µL of each inoculum was spread onto the surface. Control plates contained AgNPs without inoculum. After incubation, the MIC was recorded as the lowest concentration (µg/L) showing no visible growth^24^.

### Radiation procedure for evaluating the inhibition effects of UV, IR, and electromagnetic field on tested pathogenic strains

UVA exposure: Microbial cultures were irradiated using two 40 W fluorescent lamps (F40W/2FT/T12/6L368, Sylvania, Germany) with an emission range of 100–400 nm (UVA). The system was calibrated at the Moscow Institute of Physics and Technology (MIPT). The average irradiance at 5 cm was 220 µW/cm^2^ (2.20 × 10⁻^4^ W/cm^2^), and cultures were exposed in vitro for 10 min (600 s). The exposure dose was calculated according to the following equation: Dose (J/cm^2^) = Irradiance (W/cm^2^) × Time (s).

This yielded a UVA dose of 0.132 J/cm^2^ (132 mJ/cm^2^).

Infrared (IR) exposure: IR irradiation was performed using a 250R40/5 lamp (Sylvania, Germany) with an emission range of 700 nm–1 mm, calibrated at MIPT. The average irradiance was 155.9 mW/cm^2^ (0.1559 W/cm^2^) at 5 cm. In vivo exposure was applied for 10 min (600 s), corresponding to an IR dose of 93.5 J/cm^2^.

Electromagnetic field (EMF) exposure: The exposure was carried out on Schloder MGA_HCST_50-28 Helmholtz coil, where a uniform magnetic field of 50 Hz and 5 mT was applied in a duration of 10 min. The magnitude of the field was found out by the standard formula of Helmholtz:

$$B = {\text{8}}\mu _{0} NI\sqrt {125R}$$

where *B* is magnetic field strength (T), μ_0_ is the permeability of free space (4π × 10^−7^ H/m), N is the number of turns per coil, *I* is the applied current (A), and* R* is the coil radius (m). The system was calibrated at MIPT.

This experiment was divided into two exposure groups: (1) direct exposure of pathogens to UVA, IR or EMF, and (2) a combination exposure with synthesized AgNPs-G1 using 5 mM AgNO_3_ which was selected based on the MIC assays. Pathogens (10^8^ CFU/mL) were inoculated in the broth tubes and exposed to 10 min followed by being incubated under normal growth conditions.

### Plate count method

Following exposure to UV, IR, and 50 Hz sinusoidal EMF at 5 mT, and AgNPs, treated broth cultures (Mueller–Hinton for bacteria; Sabouraud dextrose for fungi) were serially diluted by transferring 1 mL of culture into 9 mL of sterile saline (1:10), followed by further dilutions as needed. From appropriate dilutions, 100 µL was plated onto Mueller–Hinton agar or Sabouraud dextrose agar and spread evenly using a sterile glass spreader. Plates were incubated at 37°C for 24 h (bacteria) or 28–30°C for 72 h (fungi). Colonies were counted, and viable cell concentration was calculated as CFU/mL using the formula^25^:

$${\text{CFU/mL = (Number of colonies }} \times \;{\text{Dilution factor}}){\text{ }}/{\text{ Volume plated }}\left( {{\text{mL}}} \right)$$

### Ultrastructural changes in bacteria and fungi after radiation exposure

Ultrastructural changes were examined using a JEOL TEM 140 plus (MOSCOW State Academy of Veterinary Medicine and Biotechnology_MVA). Samples were fixed, dehydrated, embedded, sectioned, and stained. TEM imaging (80–120 kV) captured micrographs, analyzed with JEOL software.

### Preparation of vaginal gel formulation

Carbopol 940 (250 g; 1.25% w/v) was gradually added to 20 L of distilled water under continuous stirring and allowed to hydrate for 2 h. The dispersion was then neutralized to pH 6.8–7.0 by the slow addition of triethanolamine (TEA; 250 g) to induce gelatin formation. 2 g of Benzoic acid was added as a preservative, and 1 g of sodium chloride was added to adjust osmolarity. The final gel was mixed until homogeneous, deaerated, and stored in sterile containers at room temperature until use. AgNPs-G1 synthesized from 5 mM AgNO_3_ were mixed with the obtained gel and coupled with IR lamp of portable infrared device. The optimal application duration on the vaginal mucosa of cattle was determined to be 10 min to ensure maximum antimicrobial efficacy.

### Statistical analysis

Data were statistically analyzed using COSTAT 2.00 software (Co-Hort Software Company). ANOVA and Fisher’s protected least significant difference (LSD) test (5% probability) were used for means separation^26^.

## Results

### The antimicrobial activity of standard antibiotic discs against pathogenic strain

Findings illustrated in Table 1 showed that the bacterial and fungal species employed in the study were sensitive to traditional antibiotics, thereby validating the experimental design for testing AgNPs and physical treatments. The most extensive antibacterial activity was exhibited by Ciprofloxacin with inhibition zone of 23–28 mm according to the tested bacterium, while Clotrimazole was the most active antifungal agent (25–29 mm).

**Table 1:** The antimicrobial activity of standard antibiotic discs against pathogenic strains expressed as mm zone of inhibition.

Standard antibiotic discs	Strains						
	***Escherichia coli***	***Pseudomonas aeruginosa***	***Staphylococcus aureus***	***Streptococcus agalactiae***	***Candida albicans***	***Aspergillus niger***	***Aspergillus*** ***fumigatus***
	**Diameter of Inhibition Zone (mm)**						
Ampicillin	14	13	19	15	-	-	-
Chloramphenicol	12	11	9	7	-	-	-
Ciprofloxacin	28	26	24	23	-	-	-
Amphortericin B	-	-	-	-	12	10	11
Clotrimazole	-	-	-	-	29	27	25
Nystatin	-	-	-	-	23	19	18

### The antimicrobial activity of probiotics with seven groups against pathogenic strains

As shown in Table 2 and Supplementary Figure 1**,** G1 showed the strongest activity (16.57 ± 5.59 mm), especially against *E. coli* (24 mm) and *P. aeruginosa* (22 mm), with moderate antifungal activity. Also, G2 exhibited strong activity (14.43 ± 5.38 mm), especially against Gram-positive bacteria (*S. aureus* and *S. agalactiae*), and reduced effectiveness against fungi. Although G3 had no activity against Gram-negative bacteria, it displayed notable antifungal effects, particularly against *C. albicans* (18.5 mm). Both G4 and G5 showed moderate activity (11.57 ± 1.51 mm and 10.14 ± 1.60 mm, respectively), mainly against *E. coli* and *P. aeruginosa*, and minimal antifungal impact. Finally, G6 and G7 exhibited the weakest activity (5.0 ± 4.73 mm and 4.57 ± 4.32 mm), with limited inhibition against bacterial strains and negligible effects on fungi. Statistical analysis established significant differences among groups (P < 0.001).

**Table 2:** The antimicrobial activity of probiotics with seven groups against pathogenic strains expressed as mm zone of inhibition.

Group	*Strains*							AVG ± SD	p
	***Escherichia coli***	***Pseudomonas aeruginosa***	***Staphylococcus aureus***	***Streptococcus agalactiae***	***Candida albicans***	***Aspergillus niger***	***Aspergillus fumigatu*s**		
	**Inhibition Zones**								
G1	24	22	19	18	12	11	10	16.57^a^ ± 5.59	< 0.001
G2	17	16	21	20	10	9	8	14.43^a^ ± 5.38	
G3	0	0	17	16	18.5	16	15.5	11.86^ab^ ± 8.16	
G4	14	12	11	10	13	11	10	11.57^ab^ ± 1.51	
G5	13	11.5	10	9	10	9	8.5	10.14^ab^ ± 1.60	
G6	10	9	8.5	7.5	0	0	0	5.0^b^ ± 4.73	
G7	9	8.5	7.5	7	0	0	0	4.57^b^ ± 4.32	
AVG	12.43^A^ ± 7.41	11.29^A^ ± 6.81	13.43^A^ ± 5.45	12.50^A^ ± 5.36	9.07^A^ ± 6.82	8.0^A^ ± 5.94	7.43^A^ ± 5.63	–	
p_**1**_	0.563								

Values represent mean inhibition zone diameters (mm) ± standard deviation.

Different superscript lowercase letters (a, b, c) within the same row indicate statistically significant differences among treatments (G1–G7) (*p* < 0.05).

Different superscript uppercase letters (A, B) within the same column indicate statistically significant differences among microbial strains (*p* < 0.05).

G1–G7 = probiotics groups (defined in Materials and Methods).

### Biosynthesis and characterization of silver nanoparticles

**Fig. 1:**
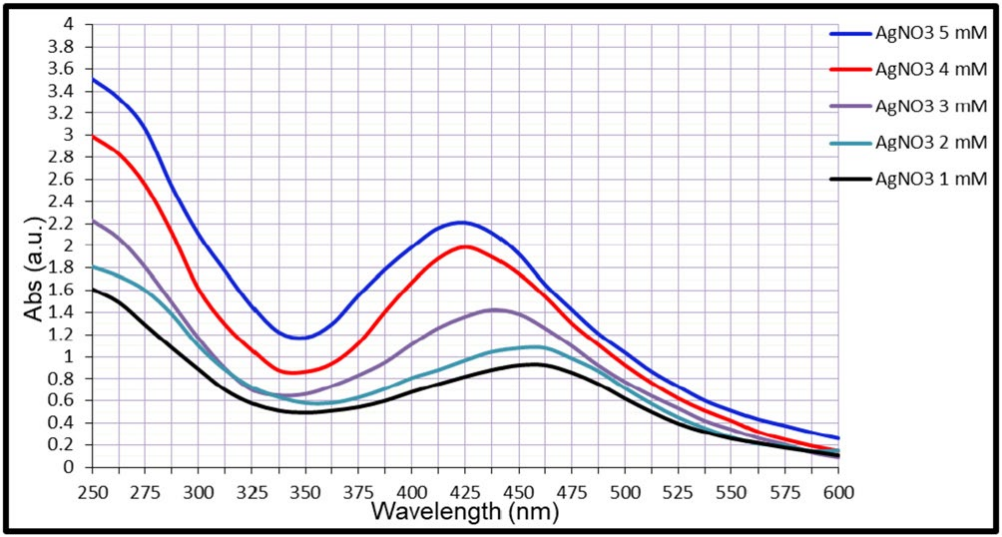
UV–Vis spectra micrograph of synthesized AgNPs-G1.

**Fig. 2:**
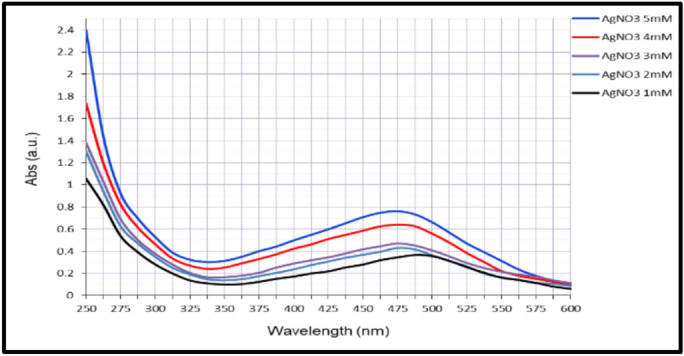
UV–Vis spectra micrograph of synthesized AgNPs-G4.

The synthesis of AgNPs was indicated by a color change from yellow to yellowish brown within 24 h after mixing AgNO₃ (1–5 mM) with cell-free probiotic filtrates. Successful nanoparticle formation occurred only in G1 and G4. UV–Vis spectroscopy (250–600 nm) confirmed synthesis of nanoparticles, with AgNPs-G1 showing a sharp peak at 425–450 nm, that indicate uniform particle size and good stability (Fig. 1). AgNPs-G4 also exhibited a broader peak at 475 nm (Fig. 2).

**Fig. 3:**
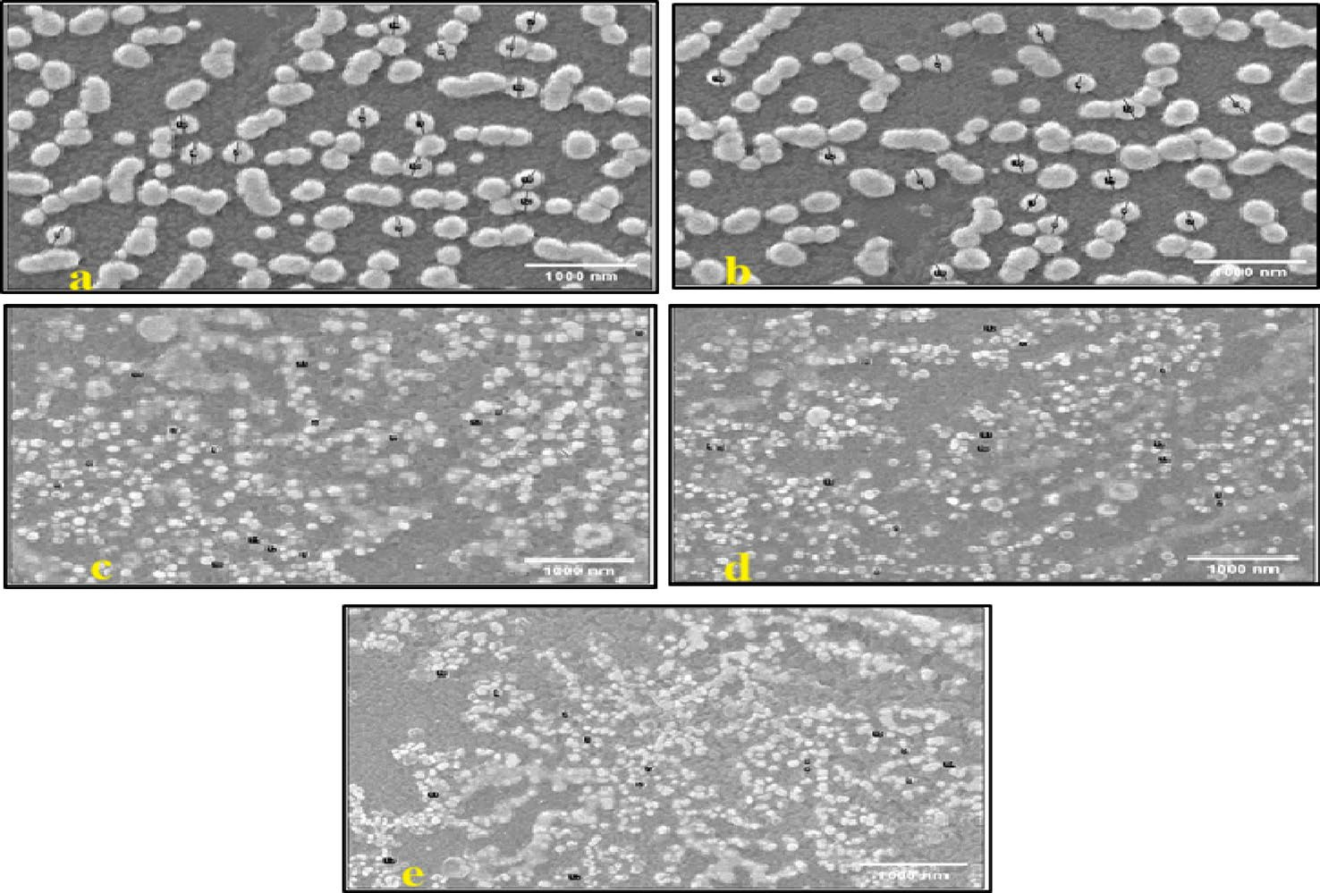
SEM images of AgNPs-G1 biosynthesized using different concentrations of AgNO_3_. (**a**) 1 mM AgNO_3_, (**b**) 2 mM AgNO_3_, (**c**) 3 mM AgNO_3_, (**d**) 4 mM AgNO_3_, (**e**) 5 mM AgNO_3._ All images were taken at 15,000 × magnification with a scale bar of 1000 nm.

**Fig. 4:**
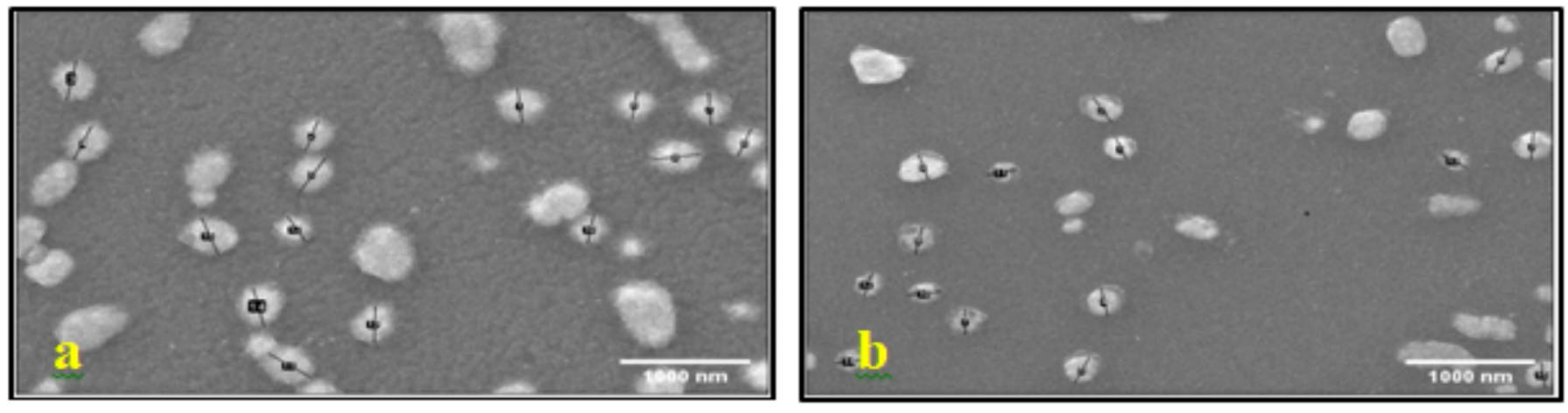
SEM images of AgNPs-G4 biosynthesized using two concentrations of AgNO_3_. (**a**) 1 mM AgNO_3_, (**b**) 5 mM AgNO_3_. All images were taken at 15,000 × magnification with a scale bar of 1000 nm.

SEM analysis showed that AgNPs-G1 were spherical, with sizes ranging from 63 to 290 nm. Larger particles were observed at 1 mM AgNO₃, while higher concentrations of 5 mM yielded smaller, more uniform nanoparticles **(**Fig. 3**)**. Likewise, AgNPs-G4 were mostly spherical with some aggregation at lower concentrations. Sizes ranged from 220 to 400 nm, decreasing progressively with higher AgNO₃ concentrations (5 mM) **(**Fig. 4**)**. It was found that both AgNPs decreased in size as the concentration was raised up. Therefore, due to the smaller average-sized particles of AgNPs-G1 synthesized using 5 mM AgNO_3_, they were chosen for further investigations.

**Fig. 5:**
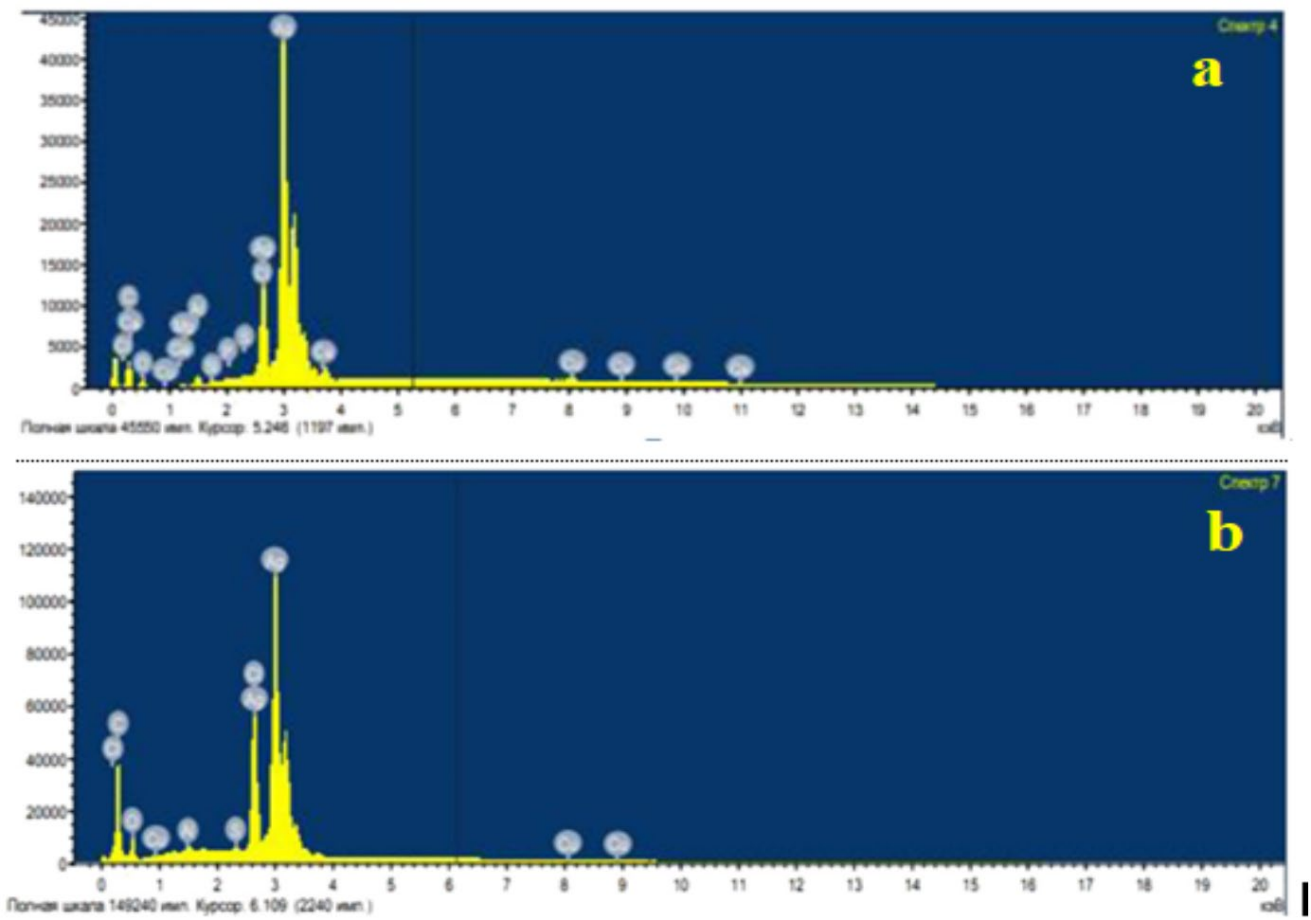
EDX spectrum of synthesized AgNPs-G1 (**a**) and AgNPs-G4 (**b**).

**Fig. 6:**
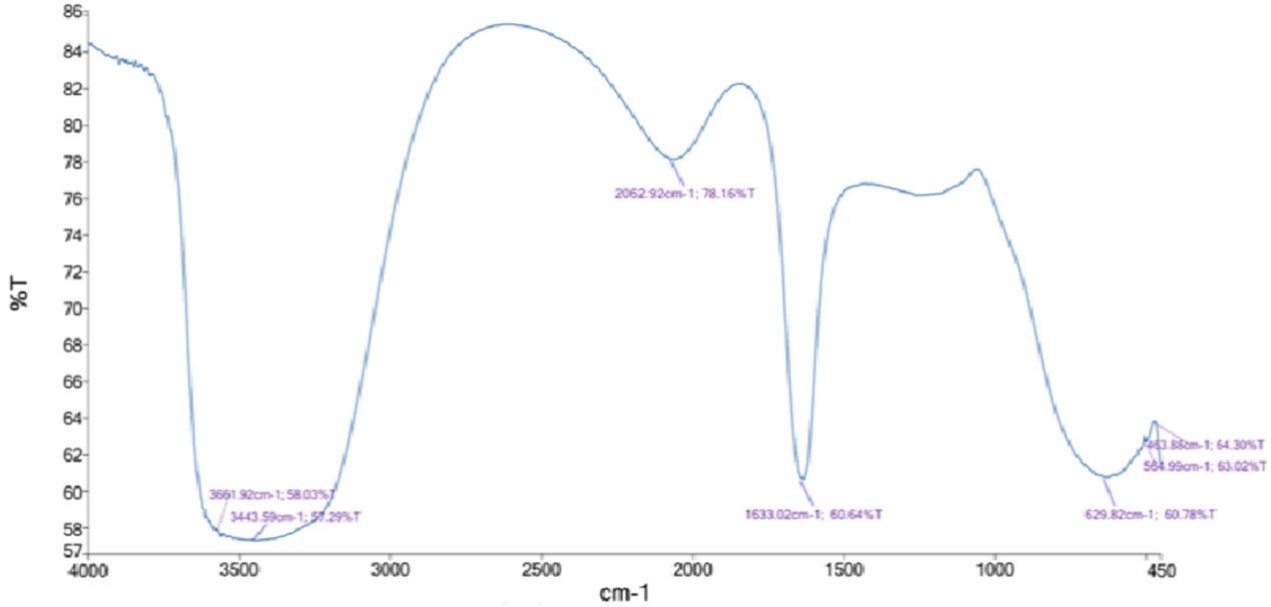
FTIR spectra of biosynthesized AgNPs-G1.

EDX analysis of AgNPs-G1 in **(**Fig. 5a**)** validates silver (Ag) as the dominant element, comprising 68.47% by weight and 24.83% by atomic percentage. Also, carbon (13.69% wt, 44.58% at) and oxygen (8.16% wt, 19.95% at) were detected; they are probably originating from organic residues or capping agents. Minor elements: Cl, Cu, Ca, S, P, Si, Al, and Mg were present in trace amounts. EDX spectra of AgNPs-G4 **(**Fig. 5b**)** confirmed the presence of silver (41.98% wt, 8.67% at), confirming nanoparticle formation. High carbon content (36.12% wt, 66.98% at) and notable oxygen levels (13.86% wt, 19.30% at) were detected. Minor elements: chlorine, aluminum, sulfur, and copper were also detected. FTIR spectrum **(**Fig. 6**)** exposed seven absorption peaks found at 3661.92, 3443.59, 2062.92, 1633.02, 629.82, 564.99, and 463.88 cm⁻^1^. These peaks correspond to the vibrational frequencies of various functional groups potentially associated with capping and stabilizing the nanoparticles.

### The antimicrobial activity of AgNPs-G1 synthesized by (1–5) mM AgNO_***3***_

Results showed a clear concentration-dependent response, with average inhibition zones increasing from 18.0 ± 7.0 mm at 1 mM to 44.29 ± 7.55 mm at 5 mM (Table 3**, **Supplementary Figure 2). This progressive increase indicates that higher concentrations of AgNPs result in stronger antimicrobial effects. *E. coli* and *S. aureus* exposed the highest sensitivity with average inhibition zones of 38.6 ± 10.6 mm and 37.20 ± 10.18 mm respectively at the highest concentration level of 5 mM while *P. aeruginosa* and *S. agalactiae* also responded well with slightly smaller zones. On the other hand, fungal strains demonstrated lower sensitivity. *Aspergillus niger* had the smallest average inhibition zone of 21.0 ± 9.9 mm, while *C. albicans* revealed moderate response with average of 24.70 ± 10.95 mm. Statistical analysis revealed a significant difference in antimicrobial activity between the five AgNPs concentrations (P < 0.001) while differences among microbial strains were not statistically significant (P₁ = 0.055). There was a trend toward variation (p = 0.055), although it was not quite statistically significant at alpha = 0.05.

**Table 3:** The antimicrobial activity of AgNPs-G1 synthesized by (1–5) mM AgNO_3_ against pathogenic strains expressed as mm zone of inhibition.

Concentrations	*Strains*							AVG ± SD	p
	***Escherichia coli***	***Pseudomonas aeruginosa***	***Staphylococcus aureus***	***Streptococcus agalactiae***	***Candida albicans***	***Aspergillus niger***	***Aspergillus fumigatu*s**		
	**Inhibition Zones**								
1 mM	25	23	25	21	11	10	11	18.0^d^ ± 7.0	< 0.001
2 mM	32	31	30	29	17	14	14	23.86^cd^ ± 8.40	
3 mM	39	37	38	36	25	20	22	31.0^bc^ ± 8.29	
4 mM	45	43	42	40	33	26	29	36.86^ab^ ± 7.47	
5 mM	52	49	51	49	37.5	35	36.5	44.29^a^ ± 7.55	
AVG	38.60^A^ ± 10.60	36.60^A^ ± 10.14	37.20^A^ ± 10.18	35.0^A^ ± 10.65	24.70^A^ ± 10.95	21.0^A^ ± 9.90	22.50^A^ ± 10.52	-	
p_**1**_	0.055								

Values are expressed as mean inhibition zone diameter (mm) ± standard deviation (SD).

Different superscript lowercase letters (a–c) within the same column indicate significant differences among AgNP concentrations (*p* < 0.05).

Different superscript uppercase letters (A–B) within the same row indicate significant differences among microbial strains (*p* < 0.05).

### The antimicrobial activity of AgNPs-G4 synthesized by (1–5) mM AgNO_3_

Results of the current study indicated an obvious concentration-dependent increase in antimicrobial activity with the inhibition zone that from 6.07 ± 5.73 mm at lower concentration (1 mM) to 18.07 ± 4.38 mm at the higher concentration (5 mM) (Table 4, Supplementary Figure 3). Both *E. coli* and *S. aureus* exhibited the maximum sensitivity at 5 mM with average inhibition zones of 17.2 ± 4.44 mm and 16.0 ± 3.95 mm, respectively. Also, *P. aeruginosa* and *S. agalactiae* displayed modest inhibition with average zones from 14.4 ± 4.29 mm to 15.7 ± 4.84 mm. On the other hand, fungal strains revealed lower sensitivity at concentrations of 1–3 mM with no inhibition zones were detected. Conversely, at 4 mM and 5 mM, weak to moderate antifungal activity emerged. Statistical analysis confirmed significant differences in the antimicrobial activity across both AgNO₃ concentrations (P < 0.006) and microbial strains (P₁ = 0.006). However, the antimicrobial potential revealed by AgNPs-G4 was lower than that reported by AgNPs-G1, therefore further investigations was performed using the later one.

**Table 4:** The antimicrobial activity of AgNPs-G4 synthesized by (1–5) mM AgNO_3_ against pathogenic strains expressed as mm zone of inhibition.

Concentrations	*Strains*							AVG ± SD	p
	***Escherichia coli***	***Pseudomonas aeruginosa***	***Staphylococcus aureus***	***Streptococcus agalactiae***	***Candida albicans***	***Aspergillus niger***	***Aspergillus fumigatu*s**		
	**Inhibition Zones**								
1 mM	12	10	11	9.5	0	0	0	6.07^c^ ± 5.73	0.006
2 mM	14	12.5	13.5	11	0	0	0	7.29^bc^ ± 6.88	
3 mM	17	15	16	14.5	0	0	0	8.93^bc^ ± 8.39	
4 mM	20	19	18.5	17	11	10	10	15.07^ab^ ± 4.53	
5 mM	23	22	21	20	14	13	13.5	18.07^a^ ± 4.38	
AVG	17.20^A^ ± 4.44	15.70^A^ ± 4.84	16.0^A^ ± 3.95	14.40^AB^ ± 4.29	5.0^B^ ± 6.93	4.60^B^ ± 6.39	4.70^B^ ± 6.55	-	
p_**1**_	0.006								

Values are expressed as mean inhibition zone diameter (mm) ± standard deviation (SD).

Different superscript lowercase letters (a–c) within the same column indicate significant differences among AgNP concentrations (*p* < 0.05).

Different superscript uppercase letters (A–B) within the same row indicate significant differences among microbial strains (*p* < 0.05).

### Minimum inhibitory concentrations of AgNPs-G1

MICs were implemented only for 5 mM AgNPs-G1 due to their enhanced antimicrobial efficacy **(**Table 5**)**. The lowest MIC was detected for *E. coli* at 25,000 µg/mL which indicates its higher sensitivity to AgNPs compared to other strains. MIC values for *P. aeruginosa*, *S. aureus*, *S. agalactiae*, and *C. albicans* are consistent at 50,000 µg/mL. On the other hand, the fungal strains like *A. niger* and *A. fumigatus* exhibit the highest MIC values at 100,000 µg/mL.

**Table 5:** Minimum inhibitory concentrations of AgNPs-G1 synthesized by 5 mM AgNO_3_ expressed as µg/mL.

Tested pathogenic strains	µg/mL
*E. coli*	25,000
*P. aeruginosa*	50,000
*S. aureus*	50,000
*S. agalactiae*	50,000
*C. albicans*	50,000
*A. niger*	100,000
*A. fumigatus*	100,000

### The antimicrobial activity of different types of radiation for inhibition the tested pathogenic strains

Table 6 reviews the different effects of IR, UV, and 50 Hz sinusoidal EMF at 5 mT radiations on the microbial growth that measured by CFU reduction, both alone and in combination with AgNPs-G1 at 5 mM AgNO₃. For *E. coli*, the IR combined with AgNPs treatment attained the highest CFU reduction of 78.9%, followed by AgNPs alone and IR alone (75.1% and 63.1% respectively) while UV and EMF alone showed limited effects (24.9% and 10.5%, respectively), which enhanced slightly when combined with AgNPs. In *P. aeruginosa*, IR + AgNPs again produced the greatest reduction (74.8%), followed by AgNPs alone (58.0%) and IR alone (55.2%). UV and EMF alone showed moderate effects (37.2% and 29.0%), which increased to 49.6% and 44.9%, respectively, when combined with AgNPs. This strain showed more resistance overall, requiring combination treatments for stronger inhibition.

**Table 6:** Bacterial/fungal cell count (CFU/ml) determined by plate count method after exposure to UV, IR, EMF and AgNPs-G1.

Treatment	*Escherichia coli*	*Pseudomonas aeruginosa*	*Staphylococcus aureus*	*Streptococcus agalactiae*	*Candida albicans*	*Aspergillus niger*	*Aspergillus fumigatu*s
Control	9.86*10^10^	1.071*10^11^	9.36*10^10^	8.80*10^10^	4.50*10^9^	5.40*10^9^	4.80*10^9^
AgNPs	2.45*10^10^	4.50*10^10^	4.56*10^10^	2.98*10^10^	2.20*10^9^	2.00*10^9^	2.50*10^9^
IR	3.64*10^10^	4.80*10^10^	5.12*10^10^	3.22*10^10^	2.90*10^9^	3.30*10^9^	3.70*10^9^
IR + AgNPs	2.08*10^10^	2.70*10^10^	1.64*10^10^	1.96*10^10^	1.80*10^9^	1.40*10^9^	1.10*10^9^
UV	7.40*10^10^	6.73*10^10^	8.86*10^10^	4.98*10^10^	3.60*10^9^	9.80*10^9^	8.70*10^9^
UV + AgNPs	6.20*10^10^	5.40*10^10^	6.24*10^10^	3.60*10^10^	2.8*10^9^	1.82*10^10^	1.75*10^10^
EMF	8.83*10^10^	7.60*10^10^	7.30*10^10^	6.32*10^10^	3.00*10^9^	8.50*10^9^	6.50*10^9^
EMF + AgNPs	7.12*10^10^	5.90*10^10^	6.65*10^10^	5.08*10^10^	2.50*10^9^	1.63*10^10^	1.39*10^10^

In the case of *S. aureus*, IR with AgNPs was most effective treatment with 82.5% reduction, while AgNPs alone achieved a reduction of 51.3% and IR alone a reduction of 45.3%. UV alone was largely ineffective (5.3%) however effectiveness improved when combined with AgNPs (33.3%). EMF alone achieved 22% reduction raised to 29% when combined with AgNPs. *S. agalactiae* exhibited the highest CFU reduction with IR with AgNPs (77.7%), followed by AgNPs alone (66.1%) and IR alone (63.4%). UV and EMF alone caused 43.4% and 28.2% reductions that increased to 59.1% and 42.3%, respectively when combined with AgNPs.

For *C. albicans*, the most effective treatment was IR combined with AgNPs of 60% reduction in CFU/mL. Also, AgNPs alone indicated strong activity with 51.1% reduction. IR radiation alone had a moderate effect with 35.6% reduction. Conversely, UV radiation alone was the least effective of 20% reduction that slightly improved when combined with AgNPs (37.8%). EMF alone reduced the fungal count by 33.3% which increased to 44.4% with AgNPs. In *A. niger*, IR + AgNPs also showed the highest effect with 74.1% reduction followed by AgNPs alone (63%). IR alone achieved a 38.9% reduction. Remarkably, both UV and EMF had adverse effects that UV alone increased fungal growth by 81.5%, and when combined with AgNPs, fungal count raised dramatically by 237%. Also, EMF alone caused a 57.4% increase, which rose to 201.9% when combined with AgNPs.

In the case of *A. fumigatus*, again IR + AgNPs achieved the highest antifungal activity reducing CFU by 77.1%, followed by AgNPs alone with 47.9% reduction. IR alone had the weakest effect among IR-based treatments of 22.9% reduction. Similar to *A. niger*, UV and EMF treatments led to an increase in fungal growth, that UV alone caused an 81.3% increase, and when combined with AgNPs caused an increase of 264.6%. EMF alone caused a 35.4% increase, and EMF + AgNPs led to a 189.6% increase.

**Fig. 7:**
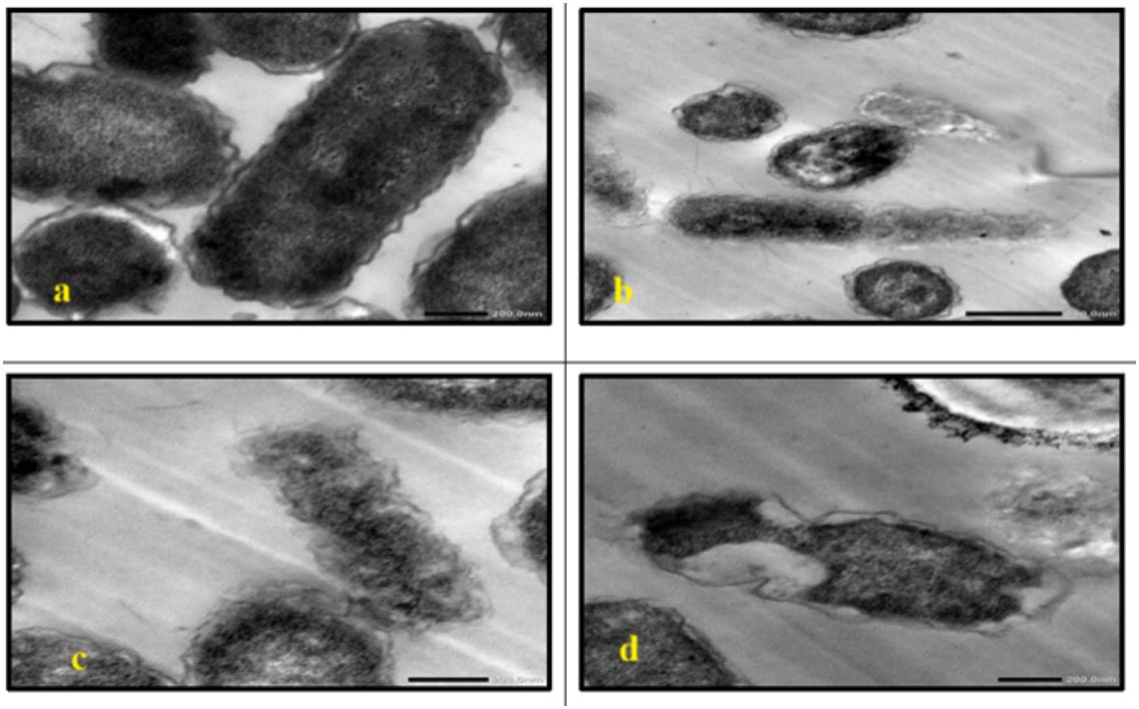
TEM micrographs of *E. coli* with different treatments. (**a**) untreated cells. (**b**) cells treated with AgNPs. (**c**) cells after exposure to IR. (**d**) cells treated with IR + AgNPs. a, c, and d images are captured at magnification: 20,000 × and scale bar: 200 nm. b is at magnification: 15,000 × and scale bar: 500 nm.

**Fig. 8:**
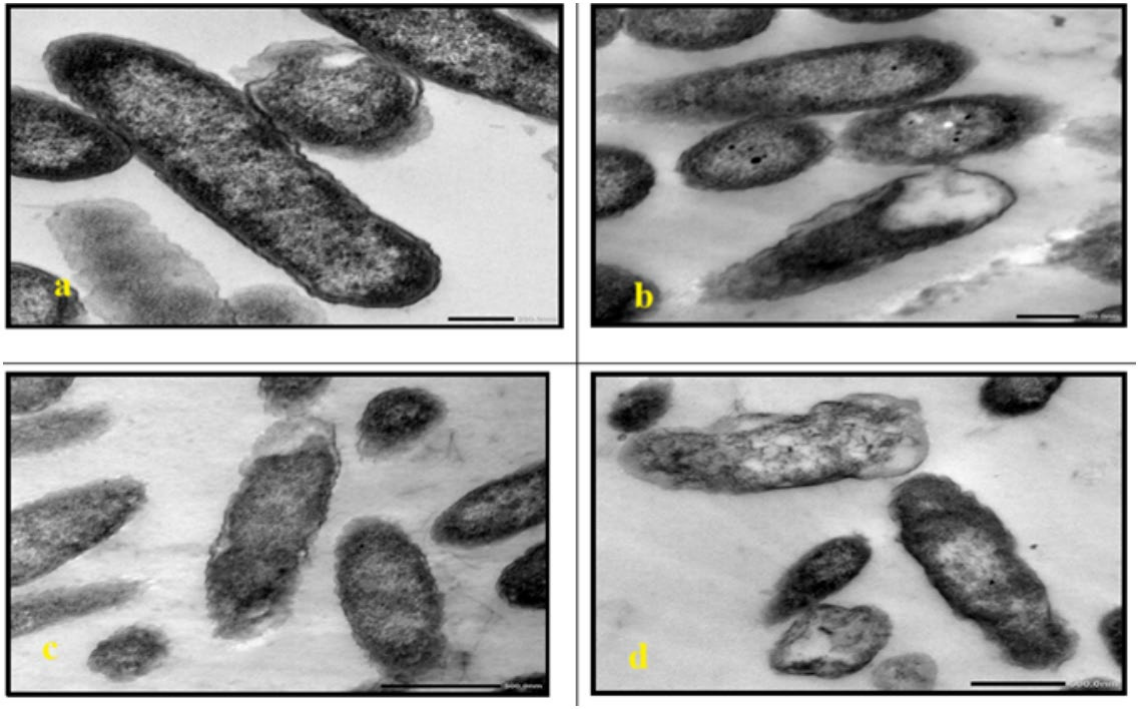
TEM micrographs of *P. aeruginosa* with different treatments. (**a**) untreated cells. (**b**) cells treated with AgNPs. (**c**) cells after exposure to IR. (**d**) cells treated with IR + AgNPs. a, b, and d images are captured at magnification:20,000 × and scale bar: 200 nm. c is at magnification: 15,000 × and scale bar: 500 nm.

**Fig. 9:**
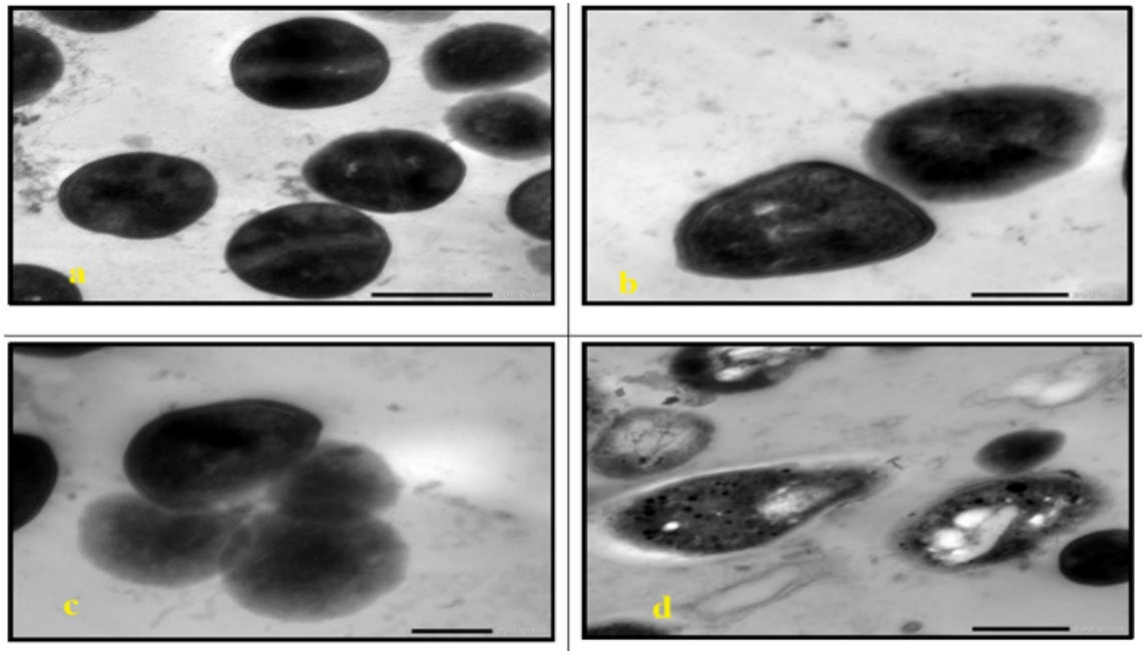
TEM micrographs of *S. aureus* with different treatments. (**a**) untreated cells. (**b**) cells treated with AgNPs. (**c**) cells after exposure to IR. (**d**) cells treated with IR + AgNPs. a and d images are captured at magnification:15,000 × and scale bar: 500 nm. b and c images are at magnification: 20,000 × and scale bar: 200 nm.

**Fig. 10:**
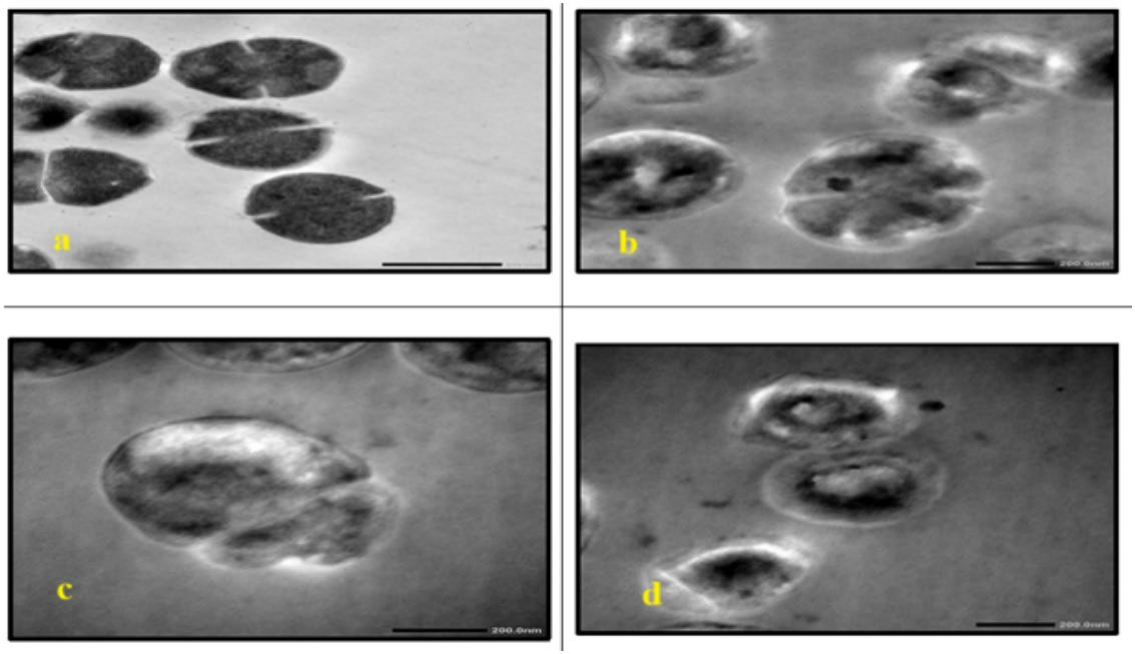
TEM micrographs of *S. agalactiae* with different treatments. (**a**) untreated cells. (**b**) cells treated with AgNPs. (**c**) cells after exposure to IR. (**d**) cells treated with IR + AgNPs. a is captured at magnification:15,000 × and scale bar: 500 nm. b, c, and d images are at magnification:20,000 × and scale bar: 200 nm.

TEM analysis of the untreated cells demonstrated a uniform microstructure, dense cytoplasm and well-organized cellular architecture **(**Fig. 7a, Fig. 13a**)**. Cells that are exposed to AgNP-G1, radiation or their combination showed remarkable morphological alterations. All tested strains showed cellular and conidial damage after treatment of IR and AgNPs. The combined treatment of IR with AgNPs had the most noticeable effect, leading to severe deformation, including collapsed protoplasts, disrupted cell walls and surface depressions. In *E. coli*, *P. aeruginosa*, *S. aureus*, and *S. agalactiae*, treatment with AgNP-G1 or IR alone caused subtle cellular effects that observed as reduced electron-dense material **(**Fig. 7b, 7c, Fig. 8b, 8c, Fig. 9c, Fig. 10b and c**)**. Moreover, slight elongation was noted in *S. aureus* cells treated with AgNP-G1 **(**Fig. 9b**)**. In opposition, *E. coli* treated with AgNP-G1 + IR displayed noticeable cytoplasmic shrinkage and ruptured cell walls **(**Fig. 7d**)**, while *P. aeruginosa* and *S. aureus* cells seemed to be irregular in shape with large and empty central regions **(**Figs. 8d, 9d**)**. *S. agalactiae* cells indicated reduced electron density and disrupted cell walls **(**Fig. 10d**)**.

**Fig. 11:**
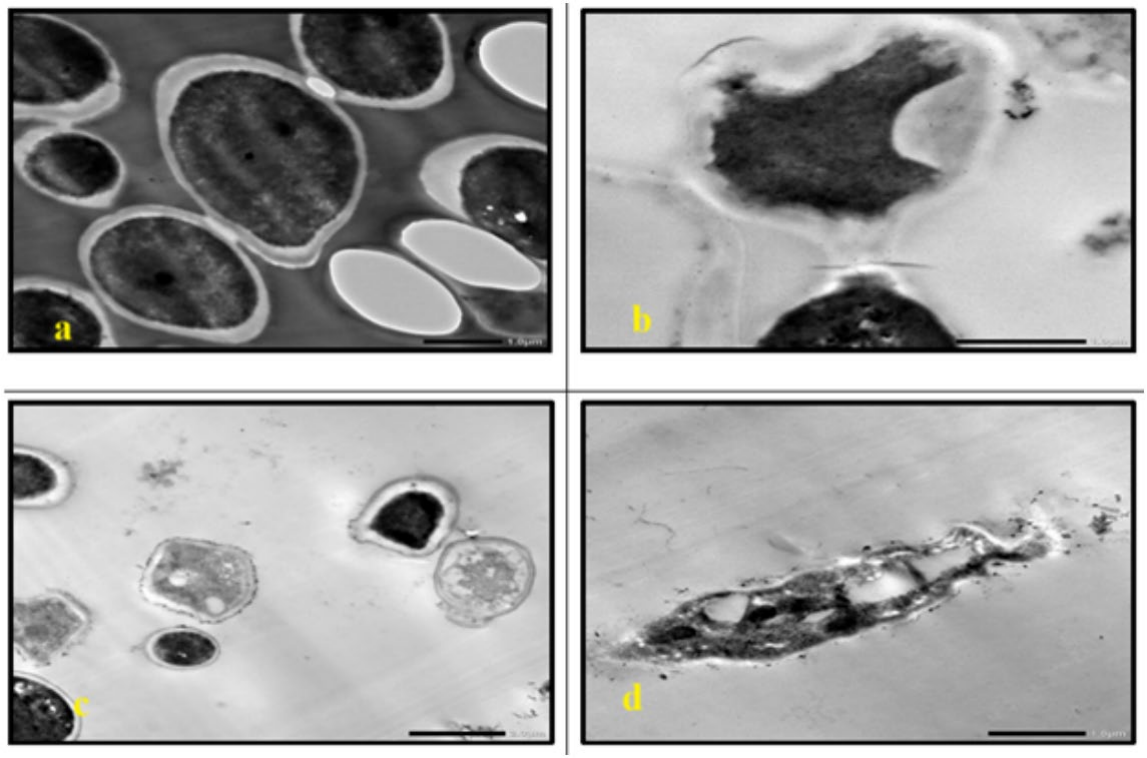
TEM micrographs of *C. albicans* with different treatments. (**a**) untreated cells. (**b**) cells treated with AgNPs. (**c**) cells after exposure to IR. (**d**) cells treated with IR + AgNPs. a, b, and d images are captured at magnification: 8000 × and scale bar: 1 µm. c is at magnification: 4000 × and scale bar: 2 µm.

**Fig. 12:**
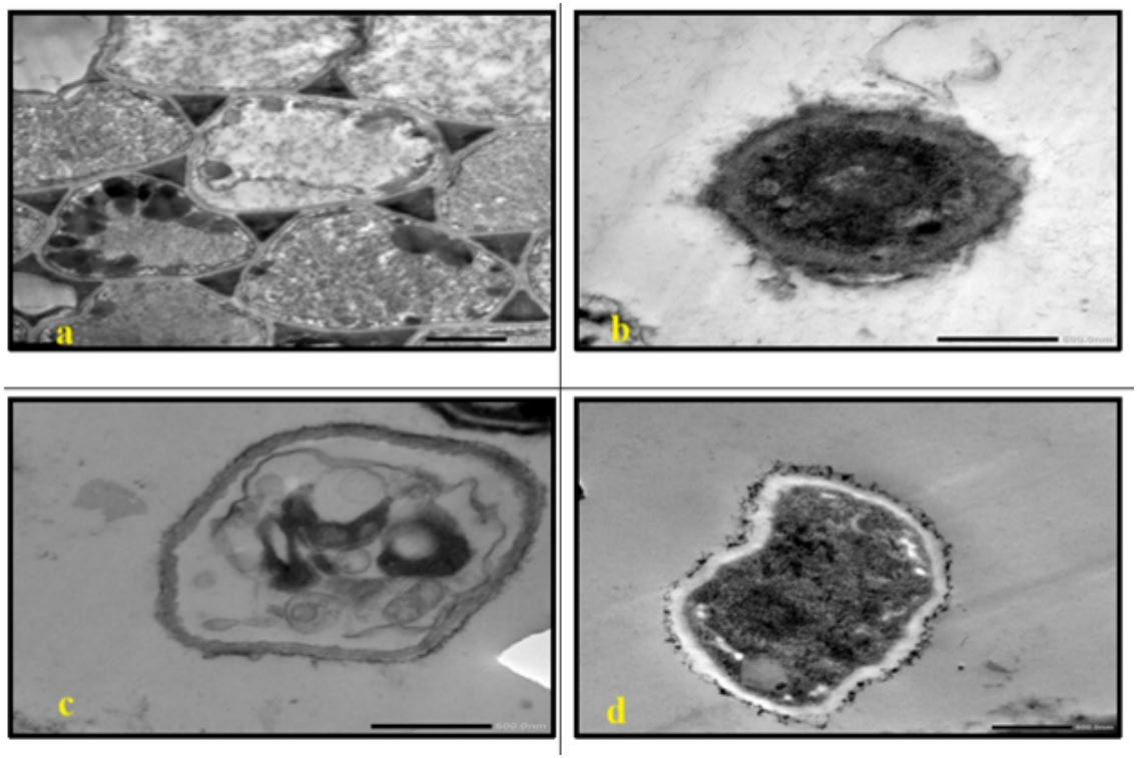
TEM micrographs of *A. niger* with different treatments. (**a**) untreated cells. (**b**) cells treated with AgNPs. (**c**) cells after exposure to IR. (**d**) cells treated with IR + AgNPs. a is captured at magnification: 4000 × and scale bar: 2 µm. b, c, and d are at magnification:15,000 × and scale bar: 500 nm.

**Fig. 13:**
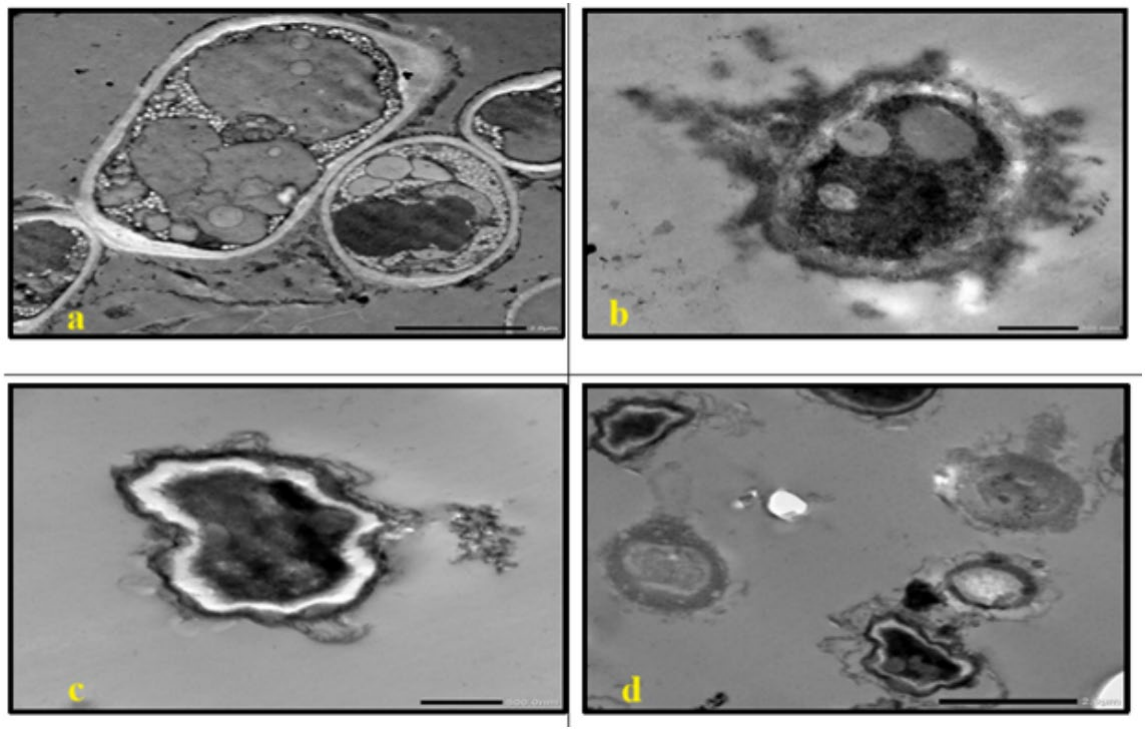
TEM micrographs of *A. fumigatus* with different treatments. (**a**) untreated cells. (**b**) cells treated with AgNPs. (**c**) cells after exposure to IR. (**d**) cells treated with IR + AgNPs. a and d are captured at magnification: 4000 × and scale bar: 2 µm. b and c are at magnification:15,000 × and scale bar: 500 nm.

**Fig. 14:**
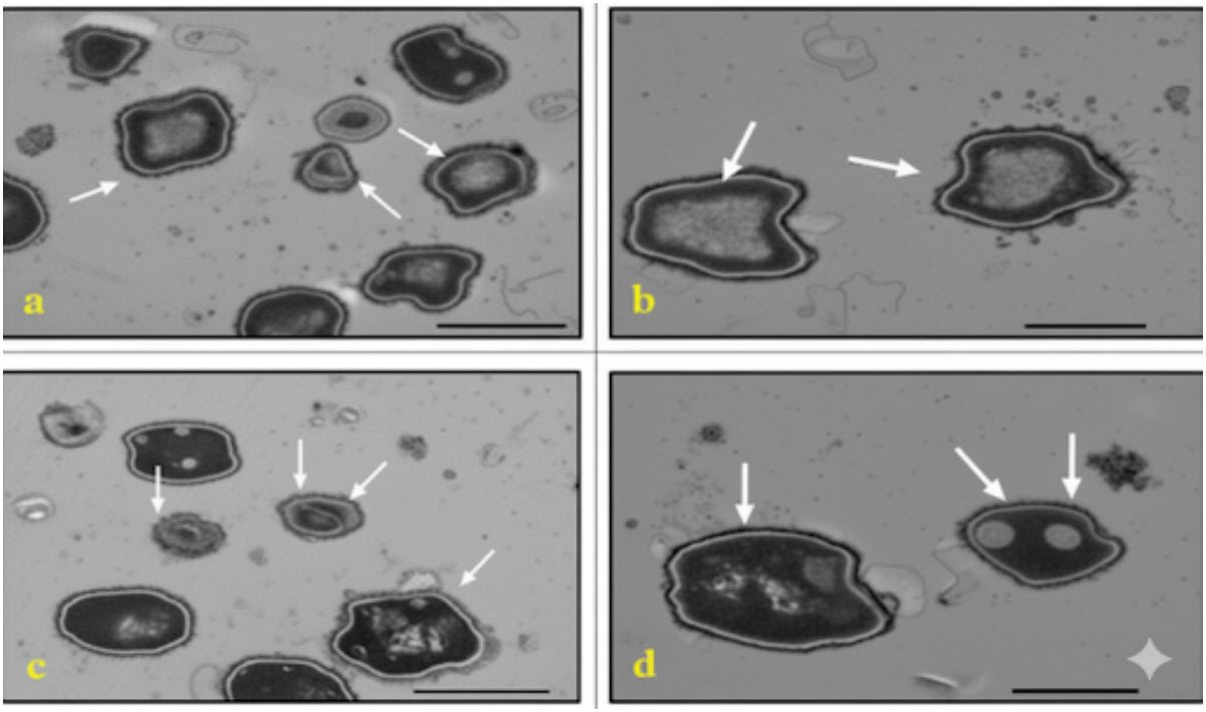
TEM micrographs of *A. niger* with AgNPs, UV and EMF. (**a**) cells exposed to UV. (**b**) cells treated with UV + AgNPs. (**c**) cells exposed to EMF. (**d**) cells treated with EMF + AgNPs. a, b, d are captured at magnification:8000 × and scale bar: 1 µm. c is at magnification: 4000 × and scale bar: 2 µm*.* Arrows indicating collapsed cell walls and surface depressions.

**Fig. 15:**
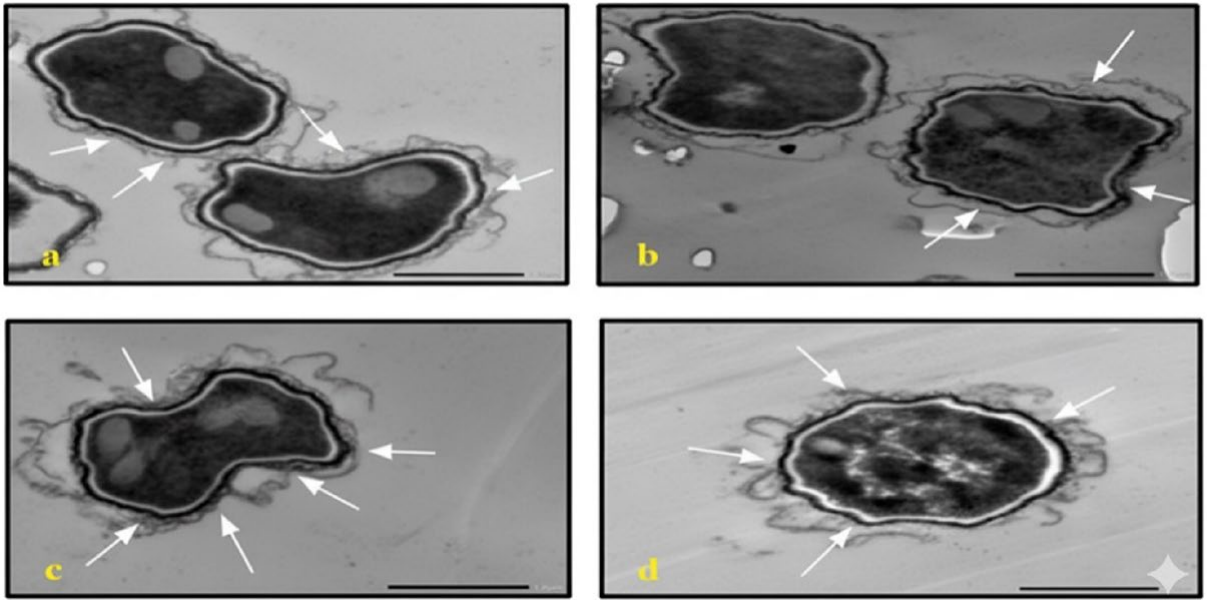
TEM micrographs of *A. fumigatus* with AgNPs, UV and EMF. (**a**) cells exposed to UV. (**b**) cells treated with UV + AgNPs. (**c**) cells exposed to EMF. (**d**) cells treated with EMF + AgNPs. All images are captured at magnification:8000 × and scale bar: 1 µm. Arrows indicating collapsed cell walls and surface depressions.

In *C. albicans*, treatment with either AgNP-G1 or IR alone resulted in abnormal cell morphology and cytoplasmic shrinkage **(**Fig. 11b, 11c**)**, whereas the combined treatment of AgNP-G1 + IR led to severe cellular damage, including leakage of intracellular contents and complete structural deformation **(**Fig. 11d**)**. Cells of *A. niger* and *A. fumigatus* treated with AgNP-G1, IR, or their combination of them demonstrated abnormal morphology, cytoplasmic shrinkage, and in the case of *A. fumigatus*, leakage of intracellular components **(**Figs. 12b–d, 13b–d**)**. Although an unexpected increase in cell count was detected in both fungal species after exposure to UV and EMF, either alone or combined with AgNPs, these treatments caused substantial damage to the conidia, evident by collapsed cell walls and surface depressions **(**Figs. 14, 15**)**.

## Discussion

The current study revealed a significant variability in the antimicrobial activity between the seven probiotic groups. For instance, G1 containing *Bacillus subtilis* VKM B-2287 and *B. licheniformis* VKM B-2414, revealed the highest inhibition zones, especially against *E. coli* and *P. aeruginosa* emphasizing its potential as a management tool against Gram-negative and multidrug-resistant bacteria^27^. Also, the strong antimicrobial activity of G2, particularly against *S. aureus* and *S. agalactiae* confirm the broad-spectrum efficacy of *Bacillus* species which consistent with the findings of^28^. Additionally, G3 consisting of *B. cereus* alone revealed no effect on Gram-negative bacteria but had remarkable antifungal activity, particularly against *C. albicans*, was likely due to its production of antifungal lipopeptides^29^. Mixed groups such as G4 (G1 + G3) and G5 (G2 + G3) exhibited moderate activity demonstrating that combining strains may not always improve effectiveness and could present competitive interactions^30^. The lowest antimicrobial activity was recorded in both G6 and G7 that contained the most diverse combinations indicating that greater strain diversity does not necessarily suppress antimicrobial metabolite production^31^.

Although all tested probiotics showed antimicrobial activity, their effects were less than conventional antibiotics (e.g., ciprofloxacin, clotrimazole). This suggests that while useful as complementary agents, they are not replacements for standard treatments. These findings support earlier observations by^32^, who reported the antimicrobial activity of several *Bacillus* strains against *E. coli*, *S. aureus*, and *P. aeruginosa*. Differences in inhibition zones may be attributed to strain-specific metabolites such as bacteriocins, surfactins, and other secondary compounds^33^. In contrast to some reports indicating that multi-strain probiotics are more effective than single strains^34^, this current study found that specific combinations, such as G1; were more effective than complex mixtures like in G7. This might reveal strain compatibility rather than just diversity. The antimicrobial zones observed resulted from mechanisms including bacteriocin production, pH modulation, and competitive exclusion^35^.

The successful synthesis of silver nanoparticles was indicated by a distinct change in color to yellowish-brown when probiotic cultures exposed to silver nitrate, a well-established visual marker of nanoparticle formation attributed to surface plasmon resonance (SPR) phenomena^36^. In biosynthesis, only probiotic strains G1 and G4 were able to synthesize AgNPs, whereas all the other strains could not produce them. This strain selectivity is probably due to strain specific extracellular differences of the two filtrates in their composition and physicochemical environment. Indeed, in microbial systems, key reducing agents, including nitrate reductase, NAD(P)H, proteins, amino acids, or polysaccharides can facilitate the conversion of Ag^+^ to Ag^0^^37^. In the absence of adequate amounts of these biomolecules, reduction may fail to commence. Further, differences in the decreased sugar, phenolic compounds, polypeptides, and capping proteins also have effects on the reduction and stabilizing of nanoparticle formation^38^. Other important factors include the physicochemical processes including pH, temperature, time of reaction which influence AgNPs synthesis, nanoparticle size and stability^39^.

UV–Vis spectroscopy revealed a strong and broad absorption peak centered on 425 nm for AgNPs-G1, which is consistent with the SPR of spherical silver nanoparticles^40^. This distinguishing peak confirmed the formation of stable metallic nanoparticles, with their shape and position affected by the nature of reducing and capping agents from the probiotic matrix that influence particle size and dispersion^41,42^.

In the current study, SEM analysis confirmed the spherical morphology of AgNPs-G1, with particle sizes ranging from 63 to 290 nm. The trend of size reduction was observed with increasing AgNO₃ concentrations which may be explained by faster nucleation rates and greater availability of silver ions for reduction. On the other hand, excessive ion concentrations can promote nanoparticle aggregation and irregular crystal formation^43^. These results align with the recent studies highlighting that optimization of precursor concentrations is essential to controlling nanoparticle shape, uniformity and functional stability in biologically mediated synthesis systems^44^.

The antibacterial efficacy of AgNPs-G1 is strongly associated with size of particles that offer higher surface area-to-volume ratio, which improves interaction with microbial cells and increases bactericidal effects^45^ demonstrated that 5 nm AgNPs exhibited a significant stronger inhibition of *E. coli* and *S. mutans* compared to larger equivalents with MIC values that declined as size decreased. Elemental analysis using energy-dispersive X-ray spectroscopy (EDX) of AgNPs-G1 showed a dominant silver peak at approximately 3 keV, confirming the presence of metallic silver. Additional minor peaks corresponding to oxygen and carbon might originate from residual biomolecules acting as capping agents that enabling nanoparticle stabilization^46^.

Infrared spectroscopic analysis clarified the role of these biomolecules in nanoparticle capping. Remarkably, absorption bands at 3661.92 and 3443.59 cm⁻^1^ are attributed to N–H and O–H stretching vibrations, suggestive of proteinaceous or other organic capping agents. For instance, the peak at 1633.02 cm⁻^1^ corresponds to amide C = O stretches, consistent with protein-linked stabilization. Lower-frequency peaks (629.82, 564.99, and 463.88 cm⁻^1^) fall within the fingerprint region, commonly related with biomolecular interactions that confirm nanoparticle formation^47,48^.

The present study demonstrated that AgNPs-G1 displayed strong and concentration-dependent antimicrobial activity. The maximum inhibition zones were observed at 5 mM AgNPs, especially against *E. coli*, outstanding even standard antibiotics. *S. aureus* also showed high sensitivity. These results align with^49^ who reported enhanced susceptibility of Gram-negative bacteria to AgNPs due to their thinner peptidoglycan layer and higher membrane permeability. Moreover, fungal strains, particularly *C. albicans*, exhibited moderate sensitivity to AgNPs, with a maximum inhibition zone of 37 mm at 5 mM. This reduced effect is likely due to the complex carbohydrate-rich structure of fungal cell walls which offers additional resistance^50^. These observations are maintained by^51^, who described the potent antibacterial and antifungal activities of AgNPs synthesized using *Lactobacillus salivarius*, with inhibition zones against fungi such as *Fusarium proliferatum* (20 mm) and *Aspergillus niger* (15 mm). Similarly,^52^ found that AgNPs synthesized from *Lacticaseibacillus rhamnosus* inhibited both Gram-positive and Gram-negative bacteria with inhibition zones up to 20 mm. A recent study by^53^ established AgNPs’ broad-spectrum efficiency, noticing a positive correlation between AgNPs concentration and the size of inhibition zone. Their silver particles which measured 10–15 nm and doped with metabolites demonstrated high stability and antimicrobial performance.

AgNPs that synthesized by G4 probiotics monitored a similar tendency revealing increased activity with raising AgNO₃ concentration. The improved antimicrobial effect of AgNPs is attributed to their ability to bind to microbial cell membranes, increasing permeability and causing structural damage^54^. Smaller nanoparticles amplify this effect^6^. The outer membrane of Gram-negative bacteria, with its porins and negatively charged lipopolysaccharides, facilitates AgNPs attachment and internalization, making these bacteria are more susceptible than Gram-positive ones^55^. Electrostatic interactions between positive charged silver ions and negative charged cell membrane constituents (e.g., phosphate, carboxyl, and amino groups) facilitate membrane penetration, disrupting the proton motive force and inducing cytoplasmic leakage^56^.

MIC is a key metric for evaluating antimicrobial effectiveness that is defined as the lowest concentration of agent that prevents visible microbial growth^57^. In this current study, AgNPs-G1 revealed the lowest MIC value was for *E. coli* that indicate its high susceptibility while *P. aeruginosa*, *S. aureus*, *S. agalactiae*, and *C. albicans* showed moderate MICs values, and *A. niger* and *A. fumigatus* were the least susceptible. These results indicate that fungal pathogens are generally more resistant to AgNPs due to their rigid chitin-rich cell walls that can hinder nanoparticle penetration. Comparatively,^58^ reported lower MIC values (4–8 µg/mL) of AgNPs against multidrug-resistant bacteria, while^59^ noted MICs ranging from 3.9 to 7.8 µg/mL against foodborne pathogens. The higher MIC values in the current study may be attributed to differences in nanoparticle synthesis methods or even larger particle sizes which can reduce bioactivity at lower concentrations.

Infrared radiation is widely applied in medical therapies and significantly enhances the antimicrobial efficiency of biosynthesized AgNPs. In our study, IR combined with AgNPs achieved over 75% bacterial reduction across most strains exceeding the effect of AgNPs or IR alone. This effect is attributed to IR-induced localized heating which improves cell membrane permeability and stimulates nanoparticle uptake, thereby amplifying oxidative stress within the cells. This mechanistic insight was confirmed by TEM images that revealed severe cellular damage including structural collapse and cytoplasmic leakage. These results align with those of^60^, who validated enhanced bacterial killing through photothermal activation of silver nanoparticles under laser irradiation, and^61^ who reported precise control over IR-induced photothermal effects in antimicrobial nanomaterials revealing similar membrane disruption and intracellular protein damage.

Conversely, UV and 50 Hz sinusoidal EMF at 5 mT radiation alone exhibited limited antimicrobial activity. Particularly with the fungal strains; *Aspergillus niger* and *A. fumigatus*; these treatments even appeared to stimulate growth. UV alone resulted in only a 5–43% reduction in bacterial viability signifying that the irradiation dose and exposure time were not enough to induce lethal DNA damage. Although UV radiation is known to cause the formation of cyclobutane pyrimidine dimers and 6–4 photoproducts, many microorganisms possess repair mechanisms such as nucleotide excision repair (NER) and photoreactivation that restore DNA integrity^62^. Ortiz-Urquiza and Keyhani^63^ claim that some fungal species have evolved strong DNA repair systems, like nucleotide excision repair and photoreactivation, that allow them to resist UV-induced damage and maintain genomic integrity. A rise in fungal colony-forming units per milliliter (CFU/mL) after exposure to UV may suggest that UV radiation functions as an environmental stressor that enhances DNA repair or causes adaptive changes. Similar results have been documented for many fungal species under UV stress, and following repeated exposure to UV radiation, mutants with improved survival traits have been observed^64^**.** Moreover, some species including spore-forming bacteria like *Bacillus* spp. and pigmented fungi like *Aspergillus* spp., exhibit natural resistance due to protective pigments like melanin or efficient DNA repair systems^64,65^, ^66^.

When UV radiation was combined with AgNPs, the antimicrobial efficacy significantly increased^67^ stated that light activation enhances Ag⁺ ion release from AgNPs and improving their bactericidal effects against antibiotic resistant bacteria which is an outcome that aligns with our enhanced bacterial reduction under UV + AgNPs treatment. Additionally, the study shows that UV exposure increases AgNPs dissolution and mobility thus elevating Ag⁺ availability and contributing to upgraded microbial inactivation as menthioned by^68^. Contrary to these results, we showed that fungus grew higher with mixed AgNP and UV treatment. This is a paradoxical reaction possibly related to a hormetic response, in which adaptive responses include an increase in antioxidant defenses, improvement in DNA repair, or alteration to cell-wall integrity pathways. These adaptive stress reactions may allow fungal cells to overcome not only combined nanoparticle- radiating but also to gain benefit through this stress as well. To the best of our knowledge, it is the first research paper that provides the readers with an account of growth restimulation of fungi exposed to AgNP + UV, which implies investigation all these issues in more detail as well as the research on strain-specific tolerance and the interaction between nanomaterials and the environment.

Exposure to electromagnetic fields can generate a range of cellular responses in microorganisms as well as altered metabolism, stress response activation and potential genetic mutations. However, 50 Hz sinusoidal EMF at 5 mT alone was not potent antimicrobial treatment in this study and even appeared to stimulate fungal growth. While the combination of EMF with AgNPs (EMF + AgNPs) enhanced bacterial reduction compared to EMF alone, it was notably less effective than the IR + AgNPs combination. Studies recommend that EMF exposure can increase reactive oxygen species production which leads to oxidative stress and possible genetic alterations. Schuermann and Mevissen^69^ stated that both radiofrequency and extremely low-frequency EMFs can modulate ROS levels in animal and cell models which may partly explain the modest antimicrobial effects observed. Furthermore,^70^ found that low-frequency EMF exposure can disrupt membrane integrity and ionic permeability, affecting microbial viability. However, EMFs may also induce adaptive responses. However,^14^ observed increased survival of *P. aeruginosa* and *E. coli* following 24-h EMF exposure when combined with subinhibitory antibiotic levels, suggesting stress adaptation rather than cell death.

Studies on filamentous fungi exposed to EMFs have reported increased enzymatic activity, sporulation and changes in secondary metabolite production indicating physiological adaptation. This may explain the enhanced growth of *A. niger* and *A. fumigatus* observed in our study. Fungi likely activate stress responses or undergo mutations that improve survival under EMF exposure^71^.

Fungal growth was further augmented by the combination of 50 Hz sinusoidal EMF at 5 mT and AgNPs, which indicated a complicated interaction between the two. No published reports exist to our knowledge of increased fungal growth when exposed to a combined 50 Hz sinusoidal EMF at 5 mT and AgNPs. Because of the surprising promotion of growth we observed under EMF + AgNPs conditions it is possible that one or perhaps both exposure conditions invoked a hormetic or adaptive response in our fungal strain. The change in the fungal cell wall or activation of efflux pumps as a result of EMF exposure, so that the cells can minimize the toxicity of AgNPs, could also be the explanation of our results. It has been known that fungi have a variety of responses to metal nanoparticles, including extracellular binding and digestion, generation of defensive biofilms, and active expulsion through nanoparticle and metal ion transport systems. The finding that the growth was higher in fungi exposed to both EMF and AgNPs may thus be due to an adaptive effect where resistance is enhanced in situations of combined stress, nanoparticle and electromagnetic irradiation. In addition, it is possible that EMFs can increase the uptake of nanoparticles by microbial cells, resulting in either their higher toxicity or, paradoxically, in increased survival because of adaptive stress responses to it^72^.

Despite this growth, TEM micrographs of cells treated with AgNPs combined with UV or EMF showed collapsed cell walls, indicating cellular damage. The increase in fungal numbers might result from their ability to utilize radiation to stimulate metabolism; a phenomenon known as radiotropism, particularly in melanized fungi, which have been shown to thrive under UV or ionizing radiation rather than being inhibited by it^73,74^.

In conclusion, the present study established the antimicrobial potential of probiotic strains, biosynthesized silver nanoparticles and their combination with physical radiation sources against a range of bacterial and fungal pathogens associated with endometritis. G1 probiotics; including *Bacillus subtilis* VKM B-2287 and *Bacillus licheniformis* VKM B-2414; exhibited the strongest antimicrobial activity among the probiotic groups. Biosynthesized AgNPs showed dose-dependent inhibitory effects, with 5 mM AgNPs achieving the highest inhibition zones especially against *E. coli* and *S. aureus*. The IR + AgNPs combination produced the most significant antimicrobial efficacy particularly for bacterial pathogens. In contrast, UV and 50 Hz sinusoidal EMF at 5 mT treatments alone were less effective and when combined with AgNPs, unexpectedly promoted fungal growth in *A. niger* and *A. fumigatus* appeared.

Finally, a formulated vaginal gel containing silver nanoparticles of 5 mM AgNO_3_ synthesized by G1 probiotics coupled with IR lamp and applied on the vaginal mucosa of cattle for 10 min for maximum antimicrobial efficacy. Prolonged exposure beyond this period is not recommended, as excessive infrared radiation may lead to adverse effects. Therefore, a controlled 10-min application is advised to achieve the best therapeutic outcome.

## Supplementary Information

Supplementary Information 1.

## Data Availability

All data generated or analyzed during this study are included in this published article. This published paper includes all the data generated or analyzed during this investigation.
