# Supplementary material for: Assessment of the antimicrobial efficacy of probiotics, biosynthesized silver nanoparticles, and their combination with physical irradiations against cattle endometritis pathogens
Source: Sci Rep. 2025 Sep 17;15:32572. doi: 10.1038/s41598-025-18623-5 (PMC12443971; doi:10.1038/s41598-025-18623-5)
Supplement: Supplementary file 1 — Supplementary Information 1. [file 41598_2025_18623_MOESM1_ESM.docx]

**Supplementary Figure 1: The inhibition zones of the tested pathogenic strains using seven groups of probiotics.**

**Supplementary Figure 2: The inhibition zones of the tested pathogenic strains using AgNPs of (1-5) mM AgNO_3_ synthesized by G1.**

**Supplementary Figure 3: The inhibition zones of the tested pathogenic strains using AgNPs of (1-5) mM AgNO_3_ synthesized by G4.**
